# A case study of a unique advanced clinical skills elective at the David Geffen School of Medicine at UCLA

**DOI:** 10.12688/mep.19397.1

**Published:** 2023-01-04

**Authors:** Michael E. Lazarus, Estebes A. Hernandez, Daniel G. Kahn, Stacey Vigallon, Christopher B. Cooper

**Affiliations:** 1 Medicine, David Greffen School of Medicine, Los Angeles, California, 90095, USA; 2 Los Angeles Audubon Society, Los Angeles, California, 90066, USA

**Keywords:** Clinical skills, visual intelligence, pattern recognition, clinical reasoning, birding, burnout mitigation

## Abstract

Proficiency in clinical examination skills upon graduation from medical school is a core competency. Over the last few decades, the ability and confidence in this fundamental and crucial skill set has declined. The motivation and interest in meticulous physical examination by recently graduated residents has also eroded. In this case study, we describe a comprehensive, innovative, and immersive advanced clinical skills elective taken during the second half of the final year of medical school for students at the David Geffen School of Medicine. The course utilizes novel approaches to inspire, refresh and consolidate essential bedside observation skills and examination techniques. This approach gives senior students the confidence and fundamental understanding of how dedication to the patient exam can improve the doctor-patient relationship, core clinical reasoning and the practice of cost-effective and evidence-based care through their careers. We describe how the integration of fine art appreciation and introductory biding techniques are used to help students hone their visual diagnostic skills. We show how this is solidified through a longitudinal series of clinical image review sessions with diagnostic reasoning principles to formulate a clear differential. Point of care ultrasound, EKG analysis, advanced cardiac auscultation and diagnostic imaging skills are integrated in a comprehensive and memorable fashion. We present this case study to inspire clinical skills teachers everywhere to replicate our methods in resurrecting the importance of physical exams for their learners. Opening their trainees’ eyes to new methods of honing their visual intelligence and developing healthy habits for stress and burnout reduction will aid the rest of their professional careers.

## Introduction

In 1994 we published an abstract ^1^ entitled, “An Advanced Course in Physical Diagnosis”. This course’s stated objective was, “[t]o refine the skills of physical diagnosis by demonstrating to students unequivocal abnormal physical signs and in doing so to resolve uncertainties that cloud clinical judgement and may lead to unnecessary laboratory and imaging studies.”

The Advanced Clinical Skills Elective has been offered to UCLA Year Four Medical Students for the past thirty years. The course was conceived as a clinical image based and patient centric advanced elective to be the consolidated review of all of clinical medicine for senior medical students prior to their graduation. Since 1994 the course has undergone significant evolution as changes in the practice of medicine have occurred but the fundamental philosophy of the sanctity of the physician-patient relationship have remained. The elective runs over a three-week period in the spring of final year. Over the course of nearly three decades, the course has evolved to include introductory bedside point-of-care ultrasound training, fine art appreciation, and birding electives. The essential threads are honing diagnostic skills by developing visual intelligence, obtaining a firsthand history, and performing a focused physical exam on real patients emphasizing deviations from the norm by observation, palpation, percussion and auscultation and then developing a pertinent differential diagnosis. In order to get to the crux of a clinical problem, this elective takes the student back to the fundamentals of medical school training, emphasizes the importance of pattern recognition and the classic, fundamental clinical skills of observation, palpation, percussion and auscultation. The course integrates data from laboratory and radiological testing as well as other modalities with the pertinent clinical information extracted from the patient, to form the basis of the clinical reasoning process. In the presence of master clinician educator preceptors, small groups of students test their hypotheses and diagnostic skills in a safe and nurturing learning environment. They are also taught that all diagnostic information is not equally helpful and that the accuracy of many clinical, laboratory and radiologic findings can be prioritized based on sensitivity, specificity and likelihood ratios, already described in the medical literature.

## Methods

All students rotate through all of these sessions and field trips over the 15 days of the course.

### Visual diagnosis

This key component of the course occurs each morning from 9:00am to 10:30am. In our medical school we have a large classroom equipped with 24 benches, each of which has two flat screen monitors. The 50 students are randomly assigned to small groups of 4–5 students, who share a bench. At the control screen, the three course chairs reveal up to ten high resolution clinical images on each group monitor per session. These clinical images were obtained and curated by the course chairs over their combined 70 years of inpatient and outpatient clinical practice in academic teaching hospital departments: general internal medicine, neurology, ophthalmology, emergency medicine, pulmonary and critical care settings. All images were obtained with patient permission or were de-identified. Most are of actual clinical signs but where appropriate, images are augmented with radiologic and micro and macro pathologic slides as a means to narrow the differential diagnosis or to clarify the clinical findings shown during the discussion. The small groups of students at each table are given 3–5 minutes to view and discuss the image and on the second attached screen at each table they generate a differential diagnosis of up to five conditions. After all groups post their differential, the course chairs choose the best differential to share with the groups on each of their screens. Each table then votes on which of the five is the most likely diagnosis. One of the course chairs then discusses the options starting with the least likely and provides confirmation of the answer. The other educators present add additional details such as clinical anecdotes, prevalence, positive and negative likelihood ratios or an interesting or amusing commentary (clinical tangent) which all help clarify and solidify the material for the learners. A running tally of which table contributed the most correct differential lists is kept and the teams/tables with the top three correct lists receive a small prize at the end of the course (usually a second-hand copy of a book from an essential reading list from the course, covering broad medical and non-medical topics). As the course progresses, the goal is to show more challenging images, common conditions with more unusual presentations as well as reducing the time allowed for discussion of the differential at each table to encourage efficiency and mastery of the subject matter.

### Highly meticulous physical exam technique demonstration and prioritization of signs by likelihood ratios ( Figure 1)

From 11am to 12pm a faculty master clinician demonstrates a physical exam to the entire group of students. These sessions are traditionally organ-system based covering cardiovascular, pulmonary, abdominal and neurologic exams. The goal of these sessions is to act as a refresher for the students from what they learnt earlier in medical school as well as to give an opportunity to provide tips on higher yield maneuvers that are supported by the literature, with high sensitivity and specificity and positive and negative likelihood ratios. The *Journal of the American Medical Association (JAMA) Rational Clinical Exam* series ^2^ is referenced as well as the book *Evidenced Based Physical Exam*, fifth edition by Steven McGee ^3^.

**Figure 1.**
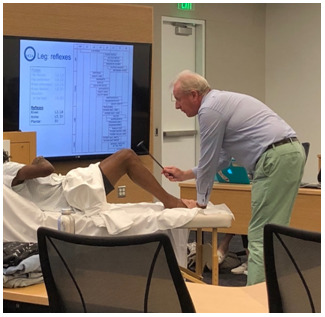
A Master clinician (C Cooper, author) demonstrates meticulous examination technique on a standardized patient.

### Real patient encounters (clinic and hospital)

Each afternoon of the course begins with clinical encounters, which consist of 90-minute inpatient and outpatient sessions that run concurrently; groups of students participate in the inpatient session while other groups attend the outpatient session. These sessions offer a core clinical experience of encounters with real patients who have pertinent histories and educationally valuable physical findings. Session one runs from 1pm–2:30pm and the second session from 2:30pm–4:00pm. Outpatient sessions take place in the medical school. Tutorial rooms are equipped with a standard outpatient exam bed, a sink, a privacy curtain, internet access and a dry erase board. The course chairs have cultivated a group of patients with chronic stable medical conditions who were identified either after admission to the inpatient teaching service, being recommended to one of the course chairs by a colleague or who are existing outpatients of the course chairs. This group of clinical teaching partners (CTP) have classic clinical exam findings such as crescendo-decrescendo systolic murmurs of aortic stenosis, the classic cutaneous signs of systemic sclerosis, or chronic stable rheumatoid arthritis. These patients are also adept at presenting their clinical histories in a concise and logical order and are comfortable interacting with, and being examined by, medical students. They are compensated at an hourly rate for their time and in some cases for travel expenses. They are also provided lunch each day and parking validation. Some CTPs will bring hard copies of their old x-rays, images of acute exacerbations of their condition (Raynaud’s Phenomenon) or their medical devices like portable oxygen canisters with oximizer pendants. Each group of students will see at least three CTP’s during their 90-minute afternoon session. Every student has the opportunity to examine the CTPs in a quiet space and receive instruction from a seasoned clinician educator every day. While these sessions are proceeding, the inpatient sessions are taking place across the street in the teaching hospital for the rest of the students. A clinician educator meets 4–5 students in the lobby and proceeds to the Emergency Department (ED), medicine ward or Intensive Care Unit (ICU). A daily list of patients with interesting clinical findings is supplied by on-service hospitalists or chief residents. Patients are similarly selected for their clinical signs and willingness to be seen by a small group of students. Some examples of patients would include those with pericardial friction rubs, neurologic signs like hemiparesis, cranial nerve lesions, clonus, muscle wasting or skin rashes, purpura and petechiae. Sometimes patients may not be in their rooms and faculty preceptors will then usually pivot to discussion of interesting radiologic findings such as the patient’s echocardiogram, electrocardiogram (EKG) or chest x-ray until the patient returns or move down their list to the next patient.

### Advanced EKG interpretation and cardiac murmur review

Three one-hour sessions from 11am–12pm are devoted to advanced electrocardiogram (EKG) analysis and auscultation of common cardiac murmurs on a simulator provided by a senior, teaching award winning faculty cardiologist. Course feedback from students over the past several years highlighted a lack of student confidence in EKG interpretation and the ability to identify common cardiac murmurs in clinical practice. We now provide three one-hour sessions for the students that cover basic and intermediate EKG interpretation with specific examples, important cardiac rhythm strip analysis of potentially life threatening arrythmias with key tips for their identification and a case-based cardiac simulation of five of the most commonly encountered cardiac murmurs in clinical practice. These sessions utilize a lifelike mannequin programmed to have the palpable clinical features of valvular disease *e.g*., bounding neck pulsations in severe aortic regurgitation as well as classic auscultatory features by stethoscope *e.g*., crescendo-decrescendo systolic murmurs, loudest in the second right intercostal space of moderate to severe aortic stenosis. We solidify this knowledge by examining patients with similar cardiac findings in the hospital and outpatient setting in many of the afternoon sessions as well. Students self-reported confidence and proficiency in cardiac auscultation has greatly improved since these additions.

### Advanced clinical imaging

This thread of this part of the course consists of three review lectures on plain radiologic film reading, reviewing computational tomography scans (CT) and a session on analyzing Magnetic resonance imaging (MRI) films by a senior radiology faculty member. Each session is based on being able to detect common radiologic findings seen in clinical medicine and emergency medicine settings. To solidify these concepts and to correlate clinical exam findings with radiologic images, the course chairs have curated a set of over 100 slides that show a physical exam finding with a radiographic image or microbiologic/pathology slide. Examples of this include images of the hands of a patient with sclerodactyly, juxtaposed with the associated radiologic features of calcinosis, flexion contractures and acro-osteolysis ^4^. These sessions take place during the afternoon for students not assigned to the clinic or inpatient rounds and are given by one of the course chairs for 90 minutes. Each student attends 2–3 of these sessions per course.

### Clinical reasoning session

Clinical reasoning is a topic that fits naturally into our course. Given that we review and expand upon many of the fundamentals of clinical medicine, we felt that adding a few sessions on this topic was essential. In the final week of the course when the students have refreshed and honed the clinical skills of history taking, physical exam and differential diagnosis formulation, we provide two sessions on clinical reasoning. We teach them how to integrate all of the information obtained into a problem representation, revising the information they gleaned from the patient with the goal of an ordered differential. In these sessions we make the students aware of all the biases that may affect their thinking and how to critically appraise their own reasoning processes. Dual process theory and metacognition principles are reviewed and expanded upon in these sessions ^5– 7^.

### Point of Care Ultrasound (POCUS) workshop and hands on practice

This thread consists of small group Point of Care Ultrasound (POCUS) sessions where students scan ultrasound simulators and each other with faculty preceptors to build on their skills of image acquisition and interpretation. Students complete assigned videos prior to the session in order to maximize hands on time. Two lectures, *Tropical medicine ultrasound* and *Pulmonary ultrasound*, are given that focus on image interpretation and clinical integration. POCUS has been demonstrated to improve diagnostic accuracy, decrease time to diagnosis, improve patient satisfaction and reduce healthcare costs, among other benefits ^8– 11^. Over the last three years, we have trained our students in POCUS to further build upon their diagnostic capabilities. This imaging technology is unique in that it brings the clinician back to the bedside and strengthens the patient physician relationship, a value that is core to this course. POCUS not only augments the physical exam, it also refines a clinician’s exam, creating more astute clinicians even in the absence of ultrasound. An example of this is evaluating the jugular venous pressure (JVP), a skill that many find elusive. When students are asked to first estimate the JVP on a patient by exam and then they are able to visually confirm the JVP, see the course of the internal jugular vein and witness hepatojugular reflux by ultrasound, their comprehension of this exam maneuver grows.

### Enhancing observational skills

***Fine art appreciation at a local museum.*** Teaching visual literacy and taking students out of the clinical setting was the basis for using fine art principles to develop observational skills ^12^. One of the recommended texts for the course is Visual Intelligence by noted art historian, Amy Herman ^13^. She utilized her degree in fine art to create the successful, “Art of Perception” program and trains thousands of professionals from Secret Service agents to medical students. We were fortunate to recruit a senior clinician educator to the course who had fine art training as an undergraduate before participating in a landmark study: “Training the Eye: Improving the Art of Physical Diagnosis” while in medical school ^14^. This study conducted at Harvard School of Medicine and the Boston Museum of Fine Art, consisted of eight paired sessions of art observation exercises with didactics that integrate fine arts concepts with physical diagnosis topics and an elective life drawing session for 24 pre-clinical students who were then compared to 34 classmates at a similar stage who did not do this training. The frequency of accurate observations on a visual skills examination was used to evaluate pre- *versus* post-course descriptions of patient photographs and art imagery. Those participants who were randomized to the art appreciation arm of the study performed significantly better than their peers in their ability to make clinical observations and in terms of their level of sophistication when describing both art and clinical images. During our course the students receive a talk given by the lead author of the above study on fine art concepts and correlation with clinical observations. On two Friday mornings of the course, field trips are organized to the UCLA Hammer Museum of Art, just a short walk from the School of Medicine. This gallery with a permanent collection of historical works and special exhibits that include local and national artists of edgier contemporary art has collaborated with us for over seven years. The morning sessions begin in the permanent collection with pairs of students describing a work of art to a fellow student who creates a drawing based on their description. Subsequent activities during the two-hour outing, include sessions in the contemporary exhibits which explore themes such as structural racism, empathy and unconscious bias; all highly relevant themes to current clinical practice.

### Bird identification at the University Botanical Garden ( Figure 2)

Another novel addition to the course is birding. Five years ago, after a close family member of one of the course chairs discovered birding and pulled the entire family into this activity, it become apparent that the skill set of the bird enthusiast is very useful for training one’s eye and cultivating visual intelligence. In birding one uses all ones’ senses to spot birds in the field. Plumage or field marks, bird sounds, behavior and the location you encounter the bird in, are all essential for identifying it in a natural environment that can be complex and dynamic. Birding can also provide a focal point for noticing, reconsidering, and understanding everyday spaces that may go unobserved, despite a high frequency of visitation. For example, features of an office courtyard or parking lot may become visible - and take on new significance - when used as birding locations. The head of educational programming for Los Angeles Audubon Society was contacted and so began a lasting collaboration with our course. She now provides an hour-long introduction to birding to the class with an emphasis on drawing. Basic bird shapes and sizes are reviewed to get the students in the mood for the Friday activity. To maintain a more manageable teaching experience, one third of the students then join her on three separate hour-long walks through the UCLA Mildred Mathias Botanical Garden starting at 8:30am. This activity usually takes place on the midpoint of the course and also serves as an opportunity to get out into nature and address how wellbeing and healthy habits can foster a long career in clinical practice. This experience was validated recently in an editorial in the *New England Journal of Medicine* where the author, who came to birding late in life, describes the cognitive requirements of birding and correlates them with the diagnostic skill required for clinical neurology ^15^. The use of birding skills to enhance clinical practice is not well studied in the medical literature but its recent increase in popularity as a pandemic pastime ^16^ has led to more articles being written including a recent publication by Koontz *et al.* in the radiology literature ^17^. Our conviction is that birding enhances observational and clinical diagnostic skills in two important ways:

**Figure 2.**
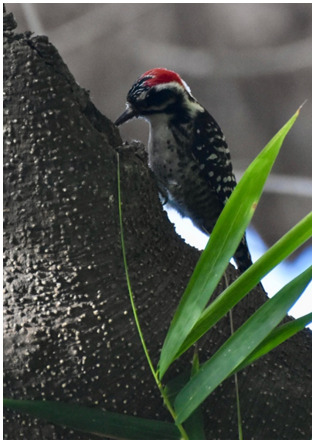
Students identify a male Nuttall’s Woodpecker based on horizontal white speckled plumage on back, red crest and the distinctive machinery-like tapping sound it produces.

1) Frequent observation of what is common enables quicker recognition leading to faster and more accurate clinical diagnosis.

2) Frequent observation of what is common enables recognition of features that are out-of-the-ordinary, and this equates to advanced clinical diagnosis.

Furthermore, the act of disengaging from work and getting out into nature are the perfect antidotes to the stresses of the hospital and clinic. Burnout mitigation, while still actively honing our visual intelligence, has been an incredible addition to the curriculum. We now receive just as many birding images as we do clinical images from our course alumni.

### Sample schedules ^18^

The scheduling of CTPs for outpatient encounters is based on the premise that the students never see the same patient during the course twice. CTPs are chosen to provide variety, but in some instances, a few recurring physical exam features appear, such as aortic stenosis murmurs, Heberden's nodes of osteoarthritis, corneal arcus, rosacea, and onychomycosis. Owing to the presence of a busy and active lung and liver transplant population at our institution, the signs of pulmonary fibrosis, spider angiomas, gynecomastia, and Dupuytren contractures are also more frequently encountered. The faculty preceptors for our course are seasoned and gifted teachers a large number of whom have won teaching awards in the medical school. While the outpatient teaching environment is relatively calm and predictable, the faculty member who does the inpatient rounds need to be efficient and pragmatic. Acute medical emergencies, code-blues, emergent testing and procedures will derail even the best planned inpatient sessions. Having the flexibility and experience to pivot on the fly is a key feature of our hospitalist faculty who often will utilize their entire inpatient list in a given afternoon. Having prior knowledge of the patient details reduces the stress or a brief teaching script is provided by course organizers.

### Modifications for distance learning during the pandemic of 2020

The course is usually offered in the last two weeks of February through the first week of March each year. In 2020, as the global Covid-19 pandemic was beginning we were able to successfully complete the course in person. We then had the luxury of an entire year to plan the 2021 course virtually. The move to all virtual instruction was seamless. The physical exam technique demonstrations were filmed and edited to maximize visualization of highly meticulous and accurate technique and also serve as a course library to be used in perpetuity. Most of our seasoned clinical teaching partners were able to master zoom, either on their own or with the aid of family members. Eighteen encounters were filmed remotely during which key history was provided to a course chair interviewer. Where possible obvious clinical exam findings were demonstrated by zooming the camera onto the relevant area. For example, sclerodactyly, digital clubbing, facial asymmetry, and arachnodactyly and hyperextensible digits in a patient with Marfan syndrome. During the course, the filmed 20-minute encounter is viewed by the group and preceptor and the CTP from the video then answers students’ questions in real time. Although not as educationally rewarding as the real thing, it is still a good teaching experience.

Teaching visual diagnosis remotely using our clinical images bank was also highly effective and students could be placed into ‘virtual breakout rooms’ in small groups to view and discuss the images and develop a differential diagnosis list. Each groups list is visible to the course chairs via a Google docs format in the main ‘zoom room’. The best differential is chosen, and students vote virtually on the best single diagnosis and faculty members provide discussion and teaching points as they would in person. Despite no hands-on instruction, the course remained highly rated by students as a valuable learning exercise. The overall course score and positive comments were not significantly different to in-person courses of prior years.

### Assessments, evaluations and learner feedback ^18^

In its early incarnations the students were expected to identify abnormal physical exam findings on hospitalized patients and were graded by faculty on examination technique. This has given way over the past two decades to the far less stressful and time-consuming visual diagnosis quiz. On the first Thursday morning of the course a 20-question test consisting of 20 clinical images is administered. Students have two minutes to answer a specific question such as “what is the diagnosis?” or ‘what is this clinical finding? At the end of the quiz the faculty review the answers with the class and they self-grade. They are requested to note their scores as on the final Thursday of the course a 30-question quiz, which is more challenging, is held and again self-graded. Students overwhelmingly do better on the second quiz. For students, it is tangible evidence that their visual intelligence has improved in three short weeks.

Course evaluations over the last 15 years have been based on 50–60 students providing a rating of 1–5 with 1 being poor and 5 being extremely effective. The overall course evaluation during this time period is 4.92/5. The most frequent, recurring comments that students have made about this course over the years are (a) this is the best clinical teaching in the whole of medical school, and (b) it should be made available to all students throughout their years in medical school ^18^.

The overall cost of running this course is quite modest. The faculty who run it are usually on their inpatient teaching rotations and integrate the additional teaching time into their daily workflow. The overall cost of reimbursing patients at an hourly rate of $35 per hour is $4,500, providing lunch daily is $1,200 and parking is $450. With a budget of under $7,000 for a 3-week course, our elective has enjoyed longevity and success as it is also cost effective and sustainable.

## Conclusion

The Advanced Clinical Skills Elective at the David Geffen School of Medicine has innovated and evolved to prepare thousands of senior medical students for residency training and beyond for over three decades. We have developed and honed a system that is holistic, innovative, cost effective and memorable. We believe some of the reasons for this courses longevity and popularity include the safe learning environment, the seniority of the students, the elective nature of the course and it’s timing to coincide with a less time intense period of medical school training. Furthermore, utilizing highly motivated senior faculty augmented by carefully chosen and skilled non-medical educator collaborations in our unique visual intelligence-based template has stood the test of time.

## Ethics statement

We confirm that we have obtained permission to use images from the faculty that are identifiable in this presentation. At the beginning of each course the students are asked verbally if images may be taken during the course for future educational use. They are reminded again when an image is about to be taken and given the option to opt out. The same request is made of all standardized patients who have signed up for student clinical skills training at the David Geffen School of Medicine. This is a case study and not considered human subject research and did not require approval from our Institutional Review Board. It is presented purely for educational purposes.

## Data Availability

Dryad: Lazarus, Michael (2022). Student comments on Advanced Clinical Skills Course https://datadryad.org/stash/dataset/doi:10.5068/D1SX1W ^18^ This project contains the following extended data: •   Data file 1. (Sample Course Schedule) •   Data file 2. (Student Comments) Data are available under the terms of the Creative Commons Zero “No rights reserved” data waiver (CC0 1.0 Public domain dedication). Access to this dataset requires registration with an IEEE account, which is free.
