# Supplementary material for: A case study of a unique advanced clinical skills elective at the David Geffen School of Medicine at UCLA
Source: MedEdPublish (2016). 2023 Jan 4;13:1. [Version 1] doi: 10.12688/mep.19397.1 (PMC9926504; doi:10.12688/mep.19397.1)
Supplement: Peer review history

# Peer review: Reviewer response for version 1

**DOI:** 10.21956/mep.20784.r32870

**Published:** 2023-02-07

**Authors:** Luann Wilkerson

**Affiliations:** 1 Dell Medical School, The University of Texas at Austin, Austin, TX, USA; 2 Emeritus at David Geffen School of Medicine at UCLA, Los Angeles, CA, USA

The article describes an elective for senior-level medical students designed to refine their skills in clinical observation, meticulous physical examination techniques, and clinical reasoning. The instructors have chosen one unusual text for the course, *Visual* *Intelligence* by Amy Herman (2016), which supports their addition more recently of art appreciation in a local museum and bird identification in the university botanical garden as tools for sharpening observational skills. The time in the garden has also triggered the inclusion of discussions on “disengaging from work and getting out into nature” as antidotes to burnout. Readers considering any of the instructional activities described in this course would benefit from the addition of student evaluations, not just of the overall effectiveness of the course, but also of the usefulness of each of the course activities as they prepare for graduate medical training.

Is the case presented with sufficient detail to be useful for teaching or other practitioners?

Yes

Is the work clearly and accurately presented and does it cite the current literature?

Yes

If applicable, is the statistical analysis and its interpretation appropriate?

Not applicable

Is the case’s background and context in sufficient detail?

Yes

Are all the source data underlying the results available to ensure full reproducibility?

Yes

Are the conclusions drawn adequately supported by the results?

Yes

Reviewer Expertise:

Curriculum design and program evaluation in medical education

I confirm that I have read this submission and believe that I have an appropriate level of expertise to confirm that it is of an acceptable scientific standard.

# Peer review: Reviewer response for version 1

**DOI:** 10.21956/mep.20784.r32839

**Published:** 2023-01-19

**Authors:** David Taylor

**Affiliations:** 1 Gulf Medical University, Ajman, United Arab Emirates

This is a really interesting article, which describes an approach to engage, or re-engage clinical students clinical skills. The article describes the educational intervention and there is a clear schedule for the intervention, and a short, but very positive selection of student evaluation comments in the associated downloads.

 The first part of the article, on the clinical skills elements, seem like normal good practice, but are doubtless valuable and important.

 I was most interested in the different ways in which the course enhancing students' observational skills. The authors give data about student satisfaction of the course, but it is not clear to me whether the student’s skills have demonstrably improved. The manuscript would have been improved by increasing (or signposting?) the references to the recent literature on "visual intelligence". All in all, though, an interesting read  about a course which could usefully be incorporated into many clinical skills course.

Is the case presented with sufficient detail to be useful for teaching or other practitioners?

Yes

Is the work clearly and accurately presented and does it cite the current literature?

Partly

If applicable, is the statistical analysis and its interpretation appropriate?

Not applicable

Is the case’s background and context in sufficient detail?

Yes

Are all the source data underlying the results available to ensure full reproducibility?

Yes

Are the conclusions drawn adequately supported by the results?

Yes

Reviewer Expertise:

Curriculum design and development

I confirm that I have read this submission and believe that I have an appropriate level of expertise to confirm that it is of an acceptable scientific standard.
